# Giving room for the existential response to serious illness: experiences with advance care planning among home-dwelling older persons admitted to acute geriatric care

**DOI:** 10.1007/s41999-026-01510-1

**Published:** 2026-06-04

**Authors:** Siri Færden Westbye, Maria Romøren, Siri Rostoft, Lisbeth Thoresen, Karin Berg Hermansen, Hege Ihle-Hansen, Trygve Johannes Lereim Sævareid, Reidar Pedersen

**Affiliations:** 1 Centre for Medical Ethics, Institute of Health and Society, Faculty of Medicine, University of Oslo, Postboks 1130, Blindern, 0318 Oslo, Norway; 2 Department of Geriatric Medicine, Oslo University Hospital, Oslo, Norway; 3 Department of Public Health Science and Interdisciplinary Health Science, Faculty of Medicine, Institute of Health and Society, University of Oslo, Oslo, Norway; 4 Department of Health Sciences, Faculty of Medicine and Health Sciences, Norwegian University of Science and Technology, Aalesund, Norway; 5 Department of Acute Medicine, Oslo University Hospital, Oslo, Norway; 6 Department of Health and Nursing Science, University of Agder, Kristiansand, Norway

**Keywords:** Advance care planning, Frailty, Community-dwelling, Autonomy, Shared decision-making, Qualitative research

## Abstract

**Purpose:**

Advance care planning (ACP) enables individuals to elicit values and preferences regarding medical treatment and care, and to record and review them before loss of capacity. In Norway as in most European countries, ACP is rarely performed. This is worrisome given the benefits of ACP, and the lack of research and understanding of the patient’s perspective, particularly in contexts where ACP is new. This paper is the first to explore how the initiation of ACP is experienced by older persons living at home with frailty who are encountering ACP for the first time, using a concept of ACP that extends beyond end-of-life decision-making.

**Methods:**

This qualitative study is part of a cluster-randomized controlled trial in which home-dwelling patients admitted to acute geriatric units were invited to participate in the initiation of ACP with their next of kin. We interviewed ten of these patients about their experiences and conducted a reflexive thematic analysis.

**Results:**

The patients reported that the initiation of ACP fostered trust and confidence, promoted emancipation and insight, enabled them to remain at home longer, opened up space for emotional and existential issues as precursors to decision-making, and promoted a more direct and honest connection with the physician.

**Conclusion:**

The initiation of ACP can be integrated into routine geriatric care and may provide important benefits for patients by filling gaps and adding new dimensions to usual care. ACP is an underused tool in communication and decisions concerning severe illness that more patients could benefit from.

ClinicalTrials.gov Identifier

NTCT05681585. Registered 03.01.23.

**Supplementary Information:**

The online version contains supplementary material available at https://doi.org/10.1007/s41999-026-01510-1.

## Key summary points

**Aim:**

To explore the experiences of older adults living with frailty who received an intervention initiated in acute geriatric units that extended beyond end-of-life decisions and included participation of next of kin.

**Findings:**

The initiation of ACP may foster trust and confidence in the physician–patient relationship, increase involvement through illness education, and give room for patients’ emotional and existential responses to illness. Participation of next of kin may also amplify patient benefit by supporting continuation of conversations after discharge, strengthening trust, and helping prevent later conflicts between relatives and clinicians when patients can no longer participate.

**Message:**

Introducing ACP earlier, during the acute admission of home-dwelling older persons living with frailty, when prognosis is still uncertain and relatives are involved, can improve the quality of communication and decision-making about serious illness by safeguarding information and involvement preferences, providing illness education, and incorporating existential concerns into conversations and decision-making about care.

**Supplementary Information:**

The online version contains supplementary material available at https://doi.org/10.1007/s41999-026-01510-1.

## Background

Appropriate care when approaching end of life depends on the person’s goals, values, and preferences [1]. Clinicians therefore have the responsibility to help the patient explore treatment options and formulate their preferences and own values, but also to give them room to process prognostic information emotionally [2].

Advance care planning (ACP) is an evidence-based clinical practice defined as a voluntary and ongoing communication process that supports individuals in understanding and sharing their personal values, life goals, and preferences regarding present and future medical care. The aim is to ensure that medical treatment aligns with patients’ wishes, particularly in the context of serious or chronic illness [3]. When practiced as a longitudinal process, ACP can also contribute to the gradual development of prognostic awareness and support patients in reflecting on what matters most to them over time [2]. Clinicians can then incorporate these values and goals into care recommendations, including decisions related to end-of-life care [2]. Furthermore, ACP can help prepare patients and families for future decision-making situations when the patient may no longer be able to participate in decisions about their care [4]. Accordingly, both international and national guidelines emphasize a process-oriented approach to ACP [3, 5–7].

Despite these recommendations, many older patients and their next of kin remain insufficiently involved in ACP [8, 9]. This is worrisome, as studies suggest that older patients generally have positive attitudes toward ACP [10, 11]. The strongest evidence for the effectiveness of ACP includes improved quality of communication, greater concordance between patients, surrogates, and clinicians regarding treatment preferences, and reduced decisional conflict [12, 13]. Nevertheless, a substantial gap persists between evidence, guideline recommendations, and clinical practice [9, 14].

Although ACP is conceptually understood as a longitudinal communication process, the intervention in this study represents the initiation of ACP during an acute hospital admission. As most participants received only a single ACP conversation, the findings primarily reflect patients’ experiences with the initial stage of ACP rather than the full ongoing process. For many participants, this conversation also constituted their first encounter with ACP, highlighting the importance of how ACP is introduced and experienced in this early phase and within a context where such conversations are still relatively new. 

From the context of end of life, a review from 2013 [10] reported that most older individuals wanted to discuss ACP, and some expressed comfort and even enthusiasm for such conversations. In contrast, a minority were reluctant [10]. A more recent review from 2018 [15] also found ambivalence among participants. Challenges in talking about ACP were largely due to the confrontation with the end of life [15]. However, patients also reported increased awareness of the seriousness of their illness as positive, and that this confrontation helped them cope with their progressive illness.

From the context of community-dwelling older persons living with frailty, a review from 2019 [16] found wide-ranging ACP views from rejection to full engagement. Most saw ACP as ‘a welcome intervention’, if conversations were conducted at the older person’s pace. The review also found that living day to day often mattered more to participants than future planning, and that relational factors and the development of trusting relationships were important.

Patients felt the acute hospital setting was an appropriate time to have ACP according to a review from 2020 [17]. Acute illness reinforced the importance of planning for the future. However, others felt that the acute setting was the wrong time, for example ‘I don’t need to talk about this today, I want to focus on getting well’.

The ACP literature mostly covers end-of-life care, including in Norway [18–22]. The only Norwegian study on patients’ views in a hospital setting also focuses on end-of-life communication for patients with life-threatening disease [23]. However, the expert consensus definition for ACP recommends it across the life continuum [3] and that it should ideally start early in adulthood [3]. Furthermore, there is a growing shift in ACP perspectives, with newer schools of thought emphasizing ongoing communication, and integration of ACP into routine care, thereby normalizing such conversations [24]. This is supported by scientific evidence that indicates a preference for early timing from the patient’s perspective [10, 15]. It is also important to study earlier contexts because ACP may be more difficult to achieve outside a palliative or a nursing home setting, and nursing homes may represent too late a stage due to the high prevalence of severe dementia [19]. Further, there is a need for more research from contexts where ACP is new [14, 25–27], and few studies have evaluated ACP interventions that include next of kin [17].

The present study is part of a cluster-randomized controlled trial aimed at implementing ACP for home-dwelling older persons living with frailty who are acutely admitted to acute geriatric hospital units.

12 units were included in the trial [28]. The trial focused on offering the initiation of ACP to all patients admitted to the acute geriatric ward, but the patients invited to the interviews were selected using specific criteria (Table 1). We selected acute geriatric units because older persons live longer with chronic illness, and acute geriatric units have spearheaded the development of more holistic approaches to the older patient in Norway. Concomitantly, ACP has become increasingly relevant beyond palliative care and oncology [7]. A unique aspect of this study is its focus on a setting where ACP is still emerging and not yet widely implemented, a situation characteristic of many countries globally [14]. The aim of this sub-study was to explore the patients’ perspective. The perspectives of next of kin are presented in a separate sub-study [29].

**Table 1:** Inclusion and exclusion criteria for patients in the qualitative sub-study

Inclusion criteria	Exclusion criteria
ACP had been conducted with next of kin and a physician and preferably a nurse^1^ Age above 70 years Home-dwelling Acutely admitted to the participating geriatric unit Clinical Frailty Scale of 4 and above [Rockwood, 2024] The physician would answer “no” to the surprise question from the Gold Standards framework proactive identification guide [Thomas, 2010] Capable of giving consent to participate in research	Expected death within 24 h Prior participation in ACP

Rockwood K, Song X, MacKnight C, et al. Clinical Frailty Scale. Accessed 13.02, 2024. https://www.legeforeningen.no/contentassets/21ef25cf569d44749573de21a8d6b043/cfs_norsk_horisontal_2021.pdf

Thomas K. The GSF prognostic indicator guidance. *End of Life Care*. 2010;4(1):62–64

^1^ Notifying next of kin afterward (e.g., by telephone) was considered acceptable

## Methods

This study uses the Reflexive Thematic Analysis Reporting Guidelines [30]. We have aimed to be as transparent as possible in describing the research process and to remain aware of prior knowledge, experiences, and potential preconceptions that could influence the research situation.

### Sample and context

The sampling strategy for the qualitative sub-study was purposeful, aiming to reach a population engaging with ACP at an earlier phase in life. The inclusion and exclusion criteria are outlined in Table 1.

All patients were recruited from the intervention arm in the trial. Patients were recruited by the physician or nurse who performed the ACP intervention, and these health professionals provided the interviewer with the patient’s contact information. The interviewer then contacted the patient, reinformed about the study, and obtained written consent. The health professionals in the participating units who delivered the intervention had received training from a local trainer using a train-the-trainer approach, as part of the larger project [28]. The interviewers involved in the qualitative sub-study were not directly involved in the ACP intervention of the trial and were not previously known to the patients.

Frailty was chosen as an inclusion criterion due to patients’ increased risk of morbidity and mortality. We recommended that next of kin participated in the intervention with the patient’s consent to capture data on the relational aspects of ACP [4, 31]. We also asked clinicians to use the surprise question “Would I be surprised if this patient died within 12 months?” [32] to identify patients in whom discussing treatment intensity and life-prolonging treatment is relevant, while simultaneously avoiding primarily dying/palliative patients [33]. We also encouraged staff to follow the five steps outlined in the ACP pocket card distributed during the implementation program (see Table 2), and an expert provided training in empathetic, person-centered communication techniques [34].

**Table 2:** Overview of the main content in the pocket card (a full version will be published in an upcoming publication from the project)

Steps	Content
1	Give a good start (explain the purpose of the conversation)
2	Information and involvement
3	Values, hopes, concerns, and if the patient has any preferences toward the end of life
4	Preferences for future healthcare
5	Summarize

The pocket card was developed based on expert definitions on ACP practice guidelines [35], the Serious illness conversation guide (SICG) [36], and an ACP-guideline tested through a trial in nursing homes in Norway [37]. In the latter trial, a two-step ACP-model was developed where patients’ preference for information and participation in decision-making are assessed first [38], before going into more substantial aspects of ACP in accordance with the expressed preferences for involvement. The pocket card (see Table 2) integrates key elements of serious illness communication (SIC) and ACP in the two-step model—emphasizing both what matters most to patients, their hopes and worries, and their preferences for information, decision-making and future treatment and care, including towards the end of life [35, 36].

In eight cases, the ACP conversation was conducted once, while in two cases it was repeated. All conversations involved one or two consultants or resident physicians, and in two cases a nurse also participated. One or more family members were present in all but one case, where the patient had no close family and only more peripheral support persons. In Norway, patients themselves designate their next of kin, and this role is by far most commonly assumed by a family member. Therefore, the absence of non-familial carers in our sample reflects typical Norwegian informal support structures rather than any exclusion criterion.

During the inclusion period, 14 patients were deemed eligible and invited, of whom 2 declined. Among the 12 who consented, one died before the interview and one withdrew, resulting in 10 completed interviews. Exact numbers of patients initially screened are unavailable. Recruitment was limited by the clinical context, as many patients were too frail or unstable to participate, and scheduling also had to accommodate next of kin. Recruitment furthermore overlapped with another sub-study involving questionnaires for patients and relatives. These factors suggest that the pool of eligible patients was small and likely close to the number invited.

Participants were recruited from four of the six geriatric units in the intervention arm. Most had a Clinical Frailty Scale (CFS) score of 4–5, reflecting a moderately frail population. The CFS score was assessed by the resident or chief physician and documented in the electronic patient record. One participant was terminally ill and died within a week after the interview.

An overview of the participants’ characteristics is presented in Table 3.

**Table 3:** Characteristics of participants

Participant number	Age	Gender	Number of days between the patient’s participation in the ACP intervention and their later participation in the qualitative interview	Number of conversations during the hospital stay	Who was involved in the ACP conversation(s) as part of the intervention
1	80	Female	6	1	Consultant, nurse and son
2	86	Male	7	1	Consultant, resident doctor, wife and grandson
3	94	Male	5	1	Consultant, resident doctor and wife
4	87	Male	18	1	Consultant, resident doctor and wife
5	84	Male	6	1	Consultant, nurse and wife
6	90	Female	5	1	Consultant, resident doctor, and two daughters
7	84	Male	7	1	Consultant and son
8	Withdrew				
9	85	Male	1	2	Resident doctor and wife
10	76	Male	2	2	Alone with resident doctor
11	88	Male	3	1	Consultant and two sons

### Data collection

The interviews were conducted between September 26, 2023, and February 2, 2024. Interviews lasted an average of 44 min (range 15–70 min). One interview was only 15 min long because cognitive impairment affected the ability to have a meaningful conversation. Six interviews were conducted in the patients’ homes, one in a nursing home, and three in the hospital. Eight interviews were conducted by SFW and three by KBH. The interview guides were semi-structured to maintain structure to answer the research question, while allowing for probing of additional themes, and enabling participants to express their views freely [39]. All the interviews were in Norwegian, and were audio recorded and transcribed.

For ethical, methodological, and practical reasons, patients and next of kin were mainly interviewed separately, as we considered that individual interviews best supported participants’ candor when discussing sensitive experiences. In the two cases where patients requested dyadic interviews, these were accommodated, and the relatives’ presence was experienced as supportive, although it added some interpretive complexity. Several of the participants also had cognitive impairment. With one exception, they were able to participate in meaningful interviews by applying techniques, such as the use of supportive prompts, simplifying questions, giving sufficient time, and using next of kin as a support. We believe this underscores the importance of including patients with cognitive impairment in ACP [4]. We present the interview guide as an online appendix to the paper (please see Additional file 1).

### Data analysis

The reflexive thematic analysis (RTA) followed Braun and Clarke’s 6-step method with attention to reflexivity throughout both the research process and reporting [40]. The approach was primarily inductive and the themes were interpreted at a latent level to capture underlying ideas and patterns. Thus, the researcher had an active role in crafting the themes in collaboration with the co-authors. The theoretical framework underpinning the thematic analysis was an experiential orientation, centering on the meanings and experiences articulated by participants [41]. The first three steps of the analysis were carried out by SFW under the supervision of RP. The first step consisted of transcribing the recorded interviews. The second step was to code interesting features of the data systematically for the whole data set, and to compile the data relevant to each code. The third step involved summarizing the codes into potential themes and organizing relevant data extracts into early thematic maps. Theme review and refinement was conducted collaboratively with the co-authors, who provided suggestions for further connections between themes, before the report was produced by SFW. Translations performed by the authors proposing the themes can introduce interpretative bias. To address this, all authors were given independent oversights of the translation process and had access to the original quotes for review. Furthermore, all authors provided feedback on the final manuscript.

## Results

This study tells the stories of patients who experienced that important aspects of patient care were not provided in usual care. They experienced the initiation of ACP as a change that both filled missing gaps and introduced new dimensions to ongoing care. We present the main themes as what was experienced as filling a gap (themes 1, 2 and 3) and adding new dimensions (themes 4 and 5). The five main themes are 1) Fostering trust and confidence, 2) Promoting emancipation and insight, 3) Enabling the patient to stay home longer, 4) Opening up for emotional and existential issues as a precursor to decision-making, and 5) Achieving a more direct and honest connection with the physician.

### Fostering trust and confidence

Most participants felt that the approach of healthcare professionals during the communication process had given them a sense of confidence. They described that the physicians and nurses were empathetic and that they felt safe talking to them. Only one participant had the impression that the personnel seemed insecure. Nevertheless, she felt that the behavior differed from what she described as a "know-it-all" attitude she was familiar with from previous visits. Family involvement was a crucial factor in participants’ experiences of safety. They argued that family members knew them well, could support them and act as witnesses. Many were routinely accompanying them to examinations, and one participant stated that his son’s presence during ACP was a ‘must’. Participants were also concerned with how decisions about treatment and care would affect their next of kin. Typical examples included spouses who had taken on most of the caregiving responsibilities.

P2: Once my state of health had been established, decisions were made. And that’s it, then I know at least one thing, that I’m not going to get better, but worse. […] So, then we have to draw the conclusion: that I must not be a burden to her.

This was also noticeable in cases where children were the primary caregiver for their parents, and the feeling of being a being a burden was addressed.

I: What thoughts have you had about where you want to live—you talked about being at home or in a care home? P6: That’s exactly what’s difficult. That’s it. I can’t rely on my kids. I: Because you are now? P6: Yes, when it comes to grocery shopping and cleaning and all sorts of things. And that’s just not okay, I think [the topic was addressed with her two children present during ACP].

In many cases, ACP contributed to reassurance that they would not become a burden and built trust regarding who should be responsible for care and how this might be shared between family members and the community services. Two participants also described that the initiation of ACP triggered a conversation that continued within the family.

### Promoting emancipation and insight

For many participants, it was the first time they had had an honest conversation about their health. At the same time, it was important for them that they were asked how much information they wanted, and that this was respected during the conversation. Those who wanted more information felt that knowing the seriousness of the situation was crucial to prepare for the future.

P10: I got a very good explanation that I have never received before. And this has been the best hospital stay I’ve ever had […] That they took themselves so much time to explain things to me, I’m impressed by that, and it stood out. It has a lot to say because—I want to know what is happening to me, so that I can prepare a little myself.

For several participants, ACP led to concrete decisions, such as stopping diagnostics or treatments.

P10: They talked about replacing my heart valves, but…

I: But you don’t want that?

P10: No. And another thing is that my quality of life isn’t very good. I’m in pain every single second that I’m awake […] That’s why I’m saying I’d rather die if things can’t change. It’s not possible to live with constant pain like this.

I: This is a very important decision you’ve made, isn’t it?

P10: Yes, it is. […] When I was asked, I didn’t need even a second to think. I said no right away. That was it. I didn’t have to think about it because I already knew, deep down, that I would never want something like that.

Promoting emancipation was also about the participants’ feeling that the doctor was taking the time to listen, explain things and involve them during the conversation. Many described this as an unusual experience with a hospital doctor. One participant who had aphasia described how her voice was often overlooked, but in her experience with ACP she felt that she had finally been heard, with family support.

P1: What bothers me is the fact that they don’t listen to everything I say. They say “it’s all right” or “it’s cleared up” when I ‘ve only said half of it because I’ve had a stroke. […] And when I say something, they put words in my mouth that I didn’t say […]. Those who don’t know me sort of ask themselves: “Is she lucid”? That’s kind of a difficult and painful question to ask, because why wouldn’t I be lucid even though I’ve been ill? And they are sort of talking over my head […] If I’ve said something, it’s not certain that they’ve understood more than half of it. But they have made up their own minds before I have said what it is. […] We [talking about her family] felt that it’s very good that the world is moving on [humorously], that something is being done, because there’s something completely wrong with the healthcare system.

Using honest language also helped inform patients and next of kin, share needs, and support both immediate and future end-of-life decisions.

P6: There was a lot of honest talk about my illness, which allowed me and [my wife] to discuss what to do. I remember saying, ‘Now we have to make a decision! We’ve been through so much—now we need to face the seriousness and talk about how we want to live.’

For two participants, however, the conversation did not hold significant meaning or lead to changes. Notably, these participants did not recall being asked specifically about their values, and their conversations were dominated by medical aspects of their condition.

P11: I wasn’t getting any healthier because of that conversation.

### Enabling the patient to stay home longer

An important consequence of the initiation of ACP for the participants was to share their personal goals. For almost all of them, the main goal was to stay home. Several participants described home conditions on the verge of breaking down, and ACP became a way to identify challenges in daily living and restore conditions that could allow them to remain safely at home. One example was a couple with severe frailty living alone, where the wife had full responsibility for caring for her husband, even though she could barely support him, resulting in repeated falls and injuries.

P3: “Eventually it was the doctor who said it—to me, and to you [turns to his wife]. You have to stop helping him. You can’t do it anymore.[Cries] […] We needed security to get life going again. But, now everything is in place [home care services and a lawyer for finances] And so we made a decision. It was special—because now we’ve actually asked to stay here [at home].”

### Opening up for emotional and existential issues as a precursor to decision-making

The participants described that their hopes and worries was explored before being asked about preferences for treatment and care. They described that this was unusual, but felt good, and for some it was a relief to have their voices and concerns heard. A participant even suggested more emphasis should have been given to the “psychological aspects”.

P7: In the conversations I’ve had, there was reason to delve deeper into things. It hasn’t really been natural to talk so much about these issues with [name of his GP and family members]. This was the first time it just naturally turned into a deeper conversation. I have the philosophy that openness… openness is the best thing in most cases. For me, it was good to talk about it.

Being able to express their existential concerns and priorities was also an important foundation for discussing the advantages and disadvantages of treatment. Several participants described that this part was often skipped in usual care.

P2: It would have been really nice to be able to talk about things like that with the people who are going to … not just cut and sew me up again […] Too many times I’ve thought about the fact that I’ve been cut a few times now, and every time I wake up, there’s no one there… I can’t talk to the guy who cut me or the girl who cut me. I don’t know who did it, and I would have liked to talk to that person.

### Achieving a more direct and honest connection with the physician

Learning as much as possible about their illness was important to participants as they navigated decisions about future care. Surprisingly, very few had previously spoken to their doctor about the state of their condition. They described that this was done carefully in this conversation, and that the physician acted as a confidant, someone who could guide them through what was likely to come. Continuity, when present, supported this direct and honest relationship, but it was not essential. Many participants simply described a different kind of contact with the physician, a closeness they more often associated with nurses.

P2: Many doctors just look in the door and then disappear again, right? And say—here. But I understand that doctors cannot get involved with all patients. That’s absolutely impossible. It’s clear that this is not the impression you get from the doctor here. […] From what I have experienced so far, I’ve had that experience with nurses, but not with doctors. I think it comes down to the chemistry you achieve with a person. There must be contact. And this contact is difficult to establish between other people in such a short time. In this case, it worked.

Honesty about health was important, but many called for sensitivity to their psychological state of mind. Two participants felt that the conversation had revolved around death and would have preferred to focus more on living day to day. However, the main pattern was that participants saw death as part of life and appreciated being able to talk about it.

P10 “I want to talk about what’s to come. I think that can be good… even if it means I’m going to bite the dust soon.”

None of the participants had previously spoken with a physician about death, and none of them found the topic difficult.

## Discussion

In this home-dwelling older population living with frailty and admitted to hospital with acute illness, we found that patients’ attitudes toward the initiation of ACP were predominantly positive, echoing previous literature [10, 11]. Although longitudinal follow-up conversations—consistent with best practice—were beyond the scope of this hospital-based intervention, participants reported that the timing, context, approach, and involvement of next of kin worked well when initiating ACP in hospital care. Beginning with an assessment of patients’ preferences for involvement [38], and then tailoring the more substantial ACP-elements accordingly, was also experienced as positive. By employing an approach that combined traditional ACP and SIC models [35, 36], this study adds to the growing body of research supporting the integration of ACP beyond palliative and oncological care [12, 24, 31, 42].

Known themes from the literature that were found are that ACP may contribute to the development of trusting relationships [10, 15], the importance of relationality in ACP [22]. Other known themes also included providing information in a way that makes patients feel empowered, respected and heard [15] and talking in an honest and straightforward manner [10, 16]. Participants experiences further reflected established advantages of ACP, such as framing prognostic information in ways that allow space for emotional processing [15], creating opportunities to explore existential concerns [43], and supporting patients in thinking about the end of life [15]. Preferences for early timing [10, 15, 17] and the focus on living day to day [10, 17, 26] were also expressed by participants in our study.

New knowledge developed in this study was that educating patients on their illness was new to the patients and gave them an agency in decision-making. We also found that factors contributing to the clarification of patients’ goals in ACP was often the impact of emotions and existential concerns leading to decision-making processes. This interview study with patients, indicates that ACP led to decisions to discontinue diagnostics and life-prolonging treatments even without a strong focus on ADs, consistent with experiences and preliminary data from the larger project [28].

In this study, the participation of next of kin seemed to amplify patient benefits by supporting continued conversations after discharge, strengthening trust, and potentially preventing conflicts between relatives and clinicians when patients are no longer able to participate. Educating patients and the next of kin about the patient’s health status, and enabling them to articulate needs also emerged as an important impact of ACP. This functioned as a precursor not only to shared decision-making in the present, but also preparing for future end-of-life decisions, consistent with recent proposals that redefine successful ACP [31]. Current hospital practice seems to favor procedures and prescriptions over communication. A recent study found that attending physicians spent a median of three minutes in the patient’s room on the morning of discharge [44]. A proposed new objective of ACP is to strengthen illness education, measured by the extent to which patient and next of kin understand the illness and prognosis, followed by the elicitation of values and goals to guide decisions [31]. Qualitative methods can identify these proximal outcomes, such as increased self‑efficacy that results when patients and families receive clear explanations of the health condition. Furthermore, introducing existential concerns into the patient-physician relationship was valued by patients and perceived as a new form of contact, fostering a more direct and honest dialog. This echoes findings from earlier studies [10, 27]. ACP could be a way to incorporate normal existential and emotional responses to illness into the biopsychosocial model, potentially reducing moral distress and burnout among healthcare personnel [45]. Partnering with patients as they navigate serious illness requires not only communicating prognostic information, but also attending to the emotions that arise from the conversation [2]. When patients do not fully comprehend their illness, prognosis, and treatment options, coupled with clinicians failing to adequately elicit patients’ values, this may exacerbate their suffering [36, 46, 47]. A facilitated ACP process can enable such communication to become more explicit, opening space for patient expression and for clinician reception and comprehension of these messages, thereby improving clinical communication and quality of care. A previous review found that physicians missed most cues (patients’ direct or indirect comments about personal aspects of their lives or their emotions) and therefore adopted behaviors that discouraged disclosure [48]. There were also examples of this in our study – even though the health personnel had been primed to address cues and concerns. Additionally, we observed that patients’ evaluation of treatment benefits might differ from healthcare professionals, as well as their perception of own quality of life. A critical goal for these patients, integral to their quality of life, was to be able to stay home as long as possible. In this study, ACP seemed to support this goal, which is recognized in the United Nations principles for older persons and reflected in various government reforms, including those in Norway [49]. This illustrates that ACP may also contribute to social innovation and the development of age-friendly societies.

Regarding timing of ACP, most participants preferred to engage in ACP early in their medical course while they were still competent, consistent with previous literature [10, 15]. Participants also felt that the hospital admission was an appropriate time, and that the hospital context reinforced the importance [26]. An implication for future research and practice is that ACP can be introduced as part of routine care when voluntariness, readiness, and a processual approach are respected, combined with clinical judgment on eligibility. Talking about death, described as a major barrier to ACP [16], was not problematic for patients in this study. Among older people living with frailty in the community, many view death as part of life [16]. One could ask whether the fear of talking about death lies more on the part of the physician than on the part of the patient.

### Strengths and limitations

The main strengths of this study are its exploration of older patients’ perspectives on ACP when introduced for the first time in a context where this is new [14, 25] and at an earlier stage—when there is still prognostic uncertainty and the patients are still home-dwelling and not in the palliative phase, and prior to loss of decision-making capacity, highlighting a patient group that has been less studied [10, 15, 16, 27], also including next of kin in the intervention [17]. This is also one of few studies on ACP where an objective measure of frailty has been used [17], which can help recognize patients at risk of deterioration, and plan realistic ACP.

Several limitations should be noted. The researchers’ roles and the study context may have influenced data collection and interpretation, introducing possible social desirability bias. Patients were invited by the same clinicians who conducted the intervention, creating a risk of selection bias. The sample may also be biased, as all participants had agreed to engage in ACP and thus may have been more comfortable with these discussions. Still, participants were encouraged to express honest views, and the data includes negative experiences. The patients declining participation may also have been reluctant to share potentially difficult or distressing experiences. In addition, the processual nature of ACP was not fully explored, as most conversations were single encounters. Nonetheless, as an exploratory study, these findings provide initial insights that can inform research with other older patients in both similar and different contexts, and serve as a foundation for future larger-scale studies.

## Conclusion

Our findings indicate that ACP can be initiated for home-dwelling older patients living with frailty during acute admissions to geriatric units, and that it is important to assess each patient’s preference for information and to respect the process-oriented and voluntary nature of ACP. We found that ACP can serve as an important tool in clinical communication and decision-making about serious illness and nearing end of life when performed through an individualized approach. Key benefits for patients included fostering trust and confidence in the physician–patient relationship, which is essential in an environment where time is scarce and continuity is challenged, as well as increasing patient involvement through illness education. Furthermore, ACP may give space for the emotional and existential responses to illness and creates opportunities for both patients and clinicians to better understand and address these concerns with the approach we used. This may assist patients in articulating their goals, and for some enable them to remain at home, which was important for many of the participants, and thus can promote both autonomy and reduce use of institutional care.

## Supplementary Information

Below is the link to the electronic supplementary material.

Supplementary file1 (DOCX 39 KB)

## Data Availability

Not applicable.

## Abbreviations

ACP: Advance care planning
AD: Advance directive
SICG: Serious illness conversation guide
SIC: Serious illness conversation

## Declarations

### Duplicate publishing

We confirm that this work is original and has not been published elsewhere, nor is it currently under consideration for publication elsewhere.
