# Supplementary material for: Giving room for the existential response to serious illness: experiences with advance care planning among home-dwelling older persons admitted to acute geriatric care
Source: Eur Geriatr Med. 2026 Jun 4;17(4):1979–89. doi: 10.1007/s41999-026-01510-1 (PMC13562197; doi:10.1007/s41999-026-01510-1)
Supplement: Supplementary file 1 [file 41999_2026_1510_MOESM1_ESM.docx]

Advance care planning for older patients admitted to hospital

INTERView guide for patients – individual interviews

**1. Experience with advance care planning**

- Who was present during the conversation? Where did the conversation take place?

- Were you invited to the conversation? Did you feel that you could say no?

- Were you asked if you wanted your carers or family members to be present? Was it important to have them with you?

- What do you think the role of carers or family members should be in such a conversation? What role do you think they should have if you are no longer able to consent to important decisions about your care?

- Did you know what advance care planning was? Do you have an understanding of it now? Explain your thoughts on it.

- How was it to talk with the doctor and the nurse (if they were present)? Did you know them? (If not, would it have been better to have someone familiar to you to be present, or is this less important?) Did you trust the person conducting the conversation?

- Did you trust the doctor and nurse during the conversation?

- Could someone other than a doctor have had the conversation?

- How did you feel about participating in this conversation? Was there anything that you found good or positive? If so, ask them to elaborate on their experience and then ask whether they believe this applies to others as well.

- Did you find it difficult to have the conversation? If so, as mentioned above, what could make it easier for you next time, and easier for everyone?

- Is it important for you to have such a conversation? What do you hope to gain from it?

**2. Content of the conversation**

- What did you talk about?

- Did the doctor speak with you about your illness and what you can expect moving forward or what probably lies ahead? Did you understand this? Did you feel uncertain about the information? If so, how was this communicated? How do you think it could have been communicated better?

- Were you asked how much information you wanted? Was this respected?

- Were you asked how involved you wanted to be in future decisions? Was this respected?

- Did you discuss what was important to you? What did you find important to talk about? Was there anything you found helpful to discuss? In what way?

- Did you talk about your hopes and concerns? How did you find this?

- Was there anything you talked about that you didn’t want to discuss?

- Was there anything you felt pressured to talk about? How detailed do you think such a conversation can be?

- Is there anything you didn’t get to say that you would have liked to share with those present?

- Is discussing the future and planning for it important, or do you prefer to talk about the “here and now”? Or both?

- Do you think it is important to talk about what is important to you?

- Did you talk about what are your thoughts or what are your wishes at the end of life?

- Do you think your personal beliefs or religion are an important part of such a conversation, or not?

**3. After the conversation**

- How did you feel afterward? What effect did it have on you (positively and negatively)?

- Do you feel more prepared for what is to come?

- Did you feel that the doctor and nurse listened to and followed up on your thoughts and feelings?

- Have you been able to talk about how it felt afterward?

**4. Timing**

- Was it the right time to have such a conversation?

- When would have been the best time for you?

- Was it the right place? If yes/no, why?

**5. Follow-up**

- Did you feel that what you said was taken into account? Provide examples if you have them.

- Do you think your wishes may change over time?

- The goal is to have several and ongoing conversations, in case your wishes and preferences change over time. What do you think about having more conversations? Have you had any? How was that? Where would you like to have more such conversations, and with whom?

- Would you take the initiative for such a conversation yourself, or do you think it should come from healthcare personnel? Do you expect it to come from healthcare personnel? Why? Please elaborate. Who do you think could follow up on such a conversation? Feel free to give examples.

- The health personnel documented the content of the conversation in your medical record. Were you aware of this, and would you like to read the note?

**6. General comments**

- Do you think other patients in similar situations should be offered an advance care planning conversation? Do you think it should be available to everyone? Would you recommend it to others? What significance do you think such conversations could have?

- Is there anything we haven’t discussed that is important for you to convey about advance care planning conversations? Alternatively, do you have any suggestions for improving how such a conversation is conducted?
